# Public Health Progress in Haiti

**DOI:** 10.4269/ajtmh.17-0347

**Published:** 2017-10-18

**Authors:** David W. Lowrance, Jordan W. Tappero, Jean Luc Poncelet, Carissa Etienne, Thomas R. Frieden, Daphne Delsoins

**Affiliations:** 1 Centers for Disease Control and Prevention, Port au Prince, Haiti;; 2 Centers for Disease Control and Prevention, Atlanta, Georgia;; 3 Pan American Health Organization, Port-au-Prince, Haiti;; 4 Pan American Health Organization, Washington, District of Columbia;; 5 Ministry of Public Health and Population, Port-au-Prince, Haiti

Although the attention of the Haitian public and international community has understandably turned to the recurrent political challenges facing the country and recovery from Hurricane Mathew, it is important that public health stakeholders take stock of progress made, remaining gaps, and fundamental public health priorities. In the past year, the global community has assessed progress toward the Millennium Development Goals (MDGs) and established new health targets under the Sustainable Development Goals framework.^1^ Haiti has made progress toward the MDGs and many of the objectives established in the aftermath of the earthquake.^1–7^ Important lessons regarding the association between various global health emergency responses, such as to human immunodeficiency virus (HIV), tuberculosis (TB), Ebola (Haitian public health professionals deployed to Guinea), and health sector resiliency have been identified.^8,9^ Yet much remains to be done to increase health service access and to reduce morbidity and mortality from preventable causes in the country. Although the public health system in Haiti has been improved since the earthquake, emergency response and health recovery funding has largely focused on new and acute threats such as Zika virus.^10^ Moreover, the health-care delivery infrastructure remains fragile, with limited coverage of primary care services, suboptimal health-care performance, and excessive health risk and vulnerability for the Haitian population. It is in this context that the Supplement, entitled “Public Health Progress in Haiti,” was developed. The reports enclosed span HIV, TB, malaria, cholera, immunizations, rabies, water, sanitation and hygiene, and lymphatic filariasis, and also address notifiable disease surveillance reporting and national laboratory capacity.^11–20^ Collectively, they appraise some of the key programs and services that have been the focus of postearthquake response and recovery planning and which have central roles informing current and future strengthening and prioritization within the health sector.

The notifiable disease surveillance system has been expanded and strengthened, facilitated by an improved public health workforce.^11^ Public health laboratory capacity has been broadened and sustained, benefitting both disease surveillance and response, and clinical services.^12^ TB case notification and detection rates have increased, in part due to expansion of TB laboratory diagnostics and other case detection efforts.^13^ HIV clinical surveillance efforts have advanced, leveraging national patient-level information systems to enable robust analyses of the clinical cascade, including retention on HIV antiretroviral treatment—an unprecedented achievement for a country facing a generalized epidemic.^14^ Collaborative research in lymphatic filariasis and malaria has enabled progress toward and underpinned commitments to elimination goals on both fronts.^15,16^ The national immunization program has advanced specific elimination goals while introducing new vaccines and strengthening surveillance and response for vaccine-preventable diseases.^17^ Novel rabies surveillance efforts have better defined burden of disease and enabled prevention services in the country.^18^ Targeted research on and innovative collaboration promoting access to safe water has informed national strategic planning for control of cholera and other diarrheal diseases.^19^ Finally, efforts to expand coverage of the oral cholera vaccine in a targeted manner have contributed to ongoing national cholera elimination efforts in the face of challenges recently heightened by Hurricane Mathew^20,21^ (Table 1).

**Table 1:** Progress in Haiti across select program areas, 2010 to 2015

Program area	Indicator	Preerthquake/2010	2015
Surveillance	Health facilities participating in national disease surveillance system	51	357
Laboratory	Diagnostic and confirmatory testing capacity in molecular, serology, parasitology, and bacteriology	2,645 tests performed at National Public Health Laboratory	20,654 tests performed at National Public Health Laboratory
TB	Case notification rates for all forms of TB	142.7/100,000	153.4/100,000
	Case notification for smear-positive pulmonary TB	85.5 cases/100,000	105.7 cases/100,000
	TB treatment success rate (WHO targets: 85% in 2010; and 90% in 2015)	79%	80%
Lymphatic filariasis	MDA	53 communes	139 communes
	Transmission assessment surveys completed	None	44 communes
			*41/44 passed and can stop MDA
Immunization	Measles, rubella and congenital rubella syndrome: progress towards elimination	Over 1,000 measles cases in 2001	Elimination declared for Measles, Rubella and congenital rubella syndrome (2014)
	Introduction of new vaccines	N/A	Pentavalent and rotavirus vaccines added to routine immunization program
	Vaccination coverage:	60% (2009)	55%
	Rotavirus (two dose)	68% (2009)	64%
	Measles-containing vaccine in children 12 months of age	65% (2009)	72% (as pentavalent)
	Three-dose diphtheria, tetanus, and pertussis		76%
	Oral polio vaccine		
Rabies	Rabies suspect animal investigations conducted	0 investigations	1,180 rabies suspect animal investigations and 75 rabid animals detected in three of 10 departments
Cholera	OCV use	OVC not used in the acute 2010 epidemic	393,688 people received two doses of OCV in three MSPP-led campaigns (2013, 2014, 2015)

MDA = mass drug administration; MSPP = Ministry of Public Health and Population; OCV = oral cholera vaccine; TB = tuberculosis; WHO = World Health Organization.

There are several relevant frameworks for considering the current status of health services and public health programs in Haiti, including those used by the Pan American Health Organization and the World Health Organization, as well as the essential public health services which have been developed to serve as a complementary paradigm with a more explicit focus on public health aspects.^22–25^ The Government of Haiti’s (GOH) health sector strategic plan, and the assessments and prioritization exercises that were performed during the early phases of emergency response planning, all remain key reference points and will be touched on throughout the various articles presented herein.^26^ Although broad and diverse, these reports do not address all important aspects of public health, such as maternal and child health, in depth. However, our hope is that, taken together, these reports will help to illuminate some of the tremendous public health gains made, and lessons learned, through the leadership of the GOH in close partnership with national and international partners.

Two issues remain central to the challenges and opportunities facing Haiti’s health sector today: financing and governance. As official data from the national health accounts vividly portray, as recently as 2004, the GOH allocated > 15% of total national expenditures to health.^27^ However, from 2005 to present, and coinciding both with the introduction of major global health financing initiatives from the Global Fund to Fight AIDS, Tuberculosis and Malaria and the U.S. President’s Emergency Plan for AIDS Relief, as well as the earthquake and cholera epidemic in 2010, there is evidence of substantial displacement of host government financing of the health sector due to external financial support, as well as the slow recovery of GOH revenues postearthquake.^28^ In the past few years, while total health spending has increased, GOH health allocations have decreased to < 5% of the national budget,^28^ despite the efforts of the Ministry of Public Health and Population (MSPP) to seek and advocate for increased health financing across other GOH institutions. Even during recent stretches of Haitian history with similar political difficulties, health allocations by the GOH remained closer to the 15% international benchmark,^29^ suggesting greater effort is needed from within the GOH, civil society, and the international community to increase emphasis on the importance of increasing and sustaining health allocations. The 2015 health financing conference convened by MSPP and health donors was a step in this direction, but the creative proposals developed need to be carried forth by the new government.^2^

Governance is another crucial public health functional domain which, although hard to define and measure, is essential to long-term public health progress. Governance and health financing are integrally intertwined, as stable financial support to hire and retain adequate human resources for health at all levels is a prerequisite for institutional capacity building and stepwise gains in sustainability. Entire units and programs within MSPP remain dependent on external donor financing to sustain adequate staffing levels and to prevent the loss of skilled workforce (“brain drain”).^30^ Such limitations can be addressed systematically with preservice educational capacity, novel health financing, and robust governance capacity within the public health sector. Only in this manner will the reputed “Republic of NGO’s”^31^ be able to establish health services and programs with a public sector backbone to promote stability and continuity, and, above all, support national efforts to provide universal access to health care. Only this will improve the health of Haitians who continue to face the worst health metrics in the Western Hemisphere.^32^

Although addressing country ownership for financing and governance is critical for ensuring that progress across public health programs in postearthquake Haiti is sustained, stronger partnerships between international and indigenous organizations are still needed to solve the greatest public health challenges. These include increasing access to clean water and improved sanitation; eliminating cholera; achieving and sustaining 90% immunization coverage for all routinely administered childhood vaccines; and ensuring supply chain for HIV antiretroviral treatment, essential cholera response commodities-like oral rehydration solution, and robust second-line drug regimens for treatment of multidrug-resistant TB.

The global health community is developing new initiatives to more effectively prepare for emerging public health threats and emergencies.^33^ Haiti’s recent experiences should both inform these efforts in other countries and regions, and ensure ongoing and legitimate partnership in the years ahead. The public health progress characterized by the reports in this Supplement should be cause for confidence and for hope that such critical efforts can and will continue even without the extraordinary impetus of disasters and emergencies, even as Haiti continues its recovery from yet another devastating storm. Given the existing and longstanding socioeconomic realities, the GOH and international donor community must be pragmatic and incremental in considering the issue of sustainability in this context.^34,35^ Focus on maintaining essential public health services, with emphasis on surveillance, laboratory capabilities, workforce capacity development, and emergency preparedness and response, while shoring up key health system building blocks such as health financing and governance, will define a pathway to maintain the hard-won momentum that has been achieved, and which should not be squandered.

## References

[1] United Nations, 2014. *Millenium Development Goals Report 2013*. Port au Prince, Haiti: Government of Haiti.

[2] Ministere de la Sante Publique et de la Population, 2015. Grands Realisations MSPP 2011–2015. Port au Prince, Haiti: Ministere de la Sante Publique et de la Population.

[3] Etienne CF Tappero JW Marston BM Frieden TR Kenyon TA Andrus JK, 2013 Cholera elimination in Hispaniola. Am J Trop Med Hyg 89: 615–616. 24106186 10.4269/ajtmh.13-0510 PMC3795089

[4] Periago MR Frieden TR Tappero JW DeCock KM Aasen B Andrus JK, 2011 Elimination of cholera transmission in Haiti and the Dominican Republic. Lancet 379: e12–e13. 10.1016/S0140-6736(12)60031-2 22240408

[5] Vertefeuille J Dowell SF Domercant JW Tappero JW, 2013 Cautious optimism on public health in post-earthquake Haiti. Lancet 381: 518–519. 10.1016/S0140-6736(13)60051-3 23332167

[6] Dowell SF Tappero JW Frieden TR, 2011 Public Health in Haiti: challenges and Progress. N Engl J Med 364: 300–301. 21219131 10.1056/NEJMp1100118

[7] Domercant JW, Guillaume FD, Marston BJ, Lowrance DW; Centers for Disease Control and Prevention, 2015 Update on progress in selected public health programsafter the 2010 earthquake and cholera epidemic: Haiti, 2014. MMWR Morb Mortal Wkly Rep. PMC4584701 25695317

[8] Ivers LC, 2011 Strengthening the health system while investing in Haiti. Am J Public Health 101: 970–971. 21493927 10.2105/AJPH.2010.300108 PMC3093291

[9] Kieny MP Dovlo D, 2015 Beyond ebola: a new agenda for resilient health systems. Lancet 385: 91–92. 25706456 10.1016/S0140-6736(14)62479-X PMC5513100

[10] Journel I, 2016 Transmission of Zika Virus – Haiti, 12 October 2015 to 10 September 2016. MMWR Morb Mortal Wkly Rep 66: 172–176. 10.15585/mmwr.mm6606a4 PMC5657860 28207688

[11] Juin S, 2017 Strengthening national disease surveillance and response: Haiti, 2010–2015. Am J Trop Med Hyg 97 (Suppl 4): 12–20. 29064361 10.4269/ajtmh.16-0948 PMC5676630

[12] Jean Louis F, 2017 Building and rebuilding: the national public health laboratory systems and services before and after the earthquake and cholera epidemic: Haiti, 2009–2015. Am J Trop Med Hyg 97 (Suppl 4): 21–27. 29064354 10.4269/ajtmh.16-0941 PMC5676632

[13] Charles M, 2017 Trends in tuberculosis case notification and treatment success, Haiti, 2010–2015. Am J Trop Med Hyg 97 (Suppl 4): 49–56. 10.4269/ajtmh.16-0863 PMC5676628 29064365

[14] Auld A Pelletier V, Shiraishi R, Deyde V, Dee J, Lowrance DW, 2017. Retention throughout the HIV care and treatment cascade: from diagnosis to antiretroviral treatment of adults and children living with HIV: Haiti, 1985–2015. Am J Trop Med Hyg 97 (Suppl 4): 57–70. 10.4269/ajtmh.17-0116 PMC5676635 29064357

[15] Lammie PJ Eberhard ML Addiss DG Won KY, Beaude Rochars M, Direny AN, Milord MD, Lafontant JG, Streit TG, 2017 Translating research into reality: elimination of lymphatic filariasis from Haiti. Am J Trop Med Hyg 97 (Suppl 4): 71–75. 10.4269/ajtmh.16-0669 PMC5676631 29064364

[16] Lemoine JF Boncy J Filler S Kachur SP Fitter D Chang MA, 2017 Haiti’s commitment to malaria elimination: progress in the face of challenges, 2010–16. Am J Trop Med Hyg 97 (Suppl 4): 43–48. 29064360 10.4269/ajtmh.16-0902 PMC5676634

[17] Tohme RA, 2017 Expansion of immunization services and strengthening vaccine Preventable diseases surveillance in Haiti, 2010–2016. Am J Trop Med Hyg 97 (Suppl 4): 28–36. 29064356 10.4269/ajtmh.16-0802 PMC5676636

[18] Wallace RM, 2017 Schrodinger’s dog: opening the box and revealing the true health impact of rabies in Haiti and recent developments in the path towards elimination, 2010–2015. Am J Trop Med Hyg 97 (Suppl 4): 76–83. 10.4269/ajtmh.16-0647 PMC5676638 29064363

[19] Patrick M, 2017 Assessment of drinking water sold from private sector kiosks in post-earthquake Port-au-Prince, Haiti. Am J Trop Med Hyg 97 (Suppl 4): 84–91. 10.4269/ajtmh.16-0692 PMC5676627 29064355

[20] Routh J, 2017 Cost evaluation of a Government-Conducted Oral Cholera Vaccination Campaign–Haiti, 2013. Am J Trop Med Hyg 97 (Suppl 4): 37–42. 29064362 10.4269/ajtmh.16-1023 PMC5676633

[21] Ivers L, 2017 Eliminating cholera transmission in Haiti. N Engl J Med 10.1056/NEJMp1614104. 10.1056/NEJMp1614104 PMC5963537 27959699

[22] World Health Organization, 2010 *Monitoring the Building Blocks of Health Systems: A Handbook of Indicators and Their Measurement Strategies*. Geneva, Switzerland: World Health Organization.

[23] World Health Organization, 2016. *Everybody’s Business: Strengthening Health Systems To Improve Health Outcomes: WHO’s Framework for Action* Available at: http://www.who.int/healthsystems/strategy/everybodys_business.pdf. Accessed April 5, 2007.

[24] Regional Office for Europe World Health Organization, 2016 *The 10 Essential Public Health Operations* Available at: http://www.euro.who.int/en/health-topics/Health-systems/public-health-services/policy/the-10-essential-public-health-operations. Accessed April 8, 2016.

[25] Centers for Disease Control and Prevention, 2015. *The Public Health System and the 10 Essential Public Health Services* Available at: http://www.cdc.gov/nphpsp/essentialservices.html. Accessed March 23, 2015.

[26] Haiti M, 2012 *Plan Directeur de Sante 2012–2022*. Port-au-Prince, Haiti: MSPP.

[27] Note d’Information Strategique, 2015 *Depenses de Santee n Haiti*. Port-au-Prince, Haiti: Ministere de la Sante Publique et de la Population.

[28] Zanotti L, 2010 Cacaphonies of aid, failed state building, and NGOs in Haiti: setting the stage for disaster, envisioning the future. Third World Q 31: 755–771. 20821882 10.1080/01436597.2010.503567

[29] World Health Organization, 2011. *The Abuja Declaration: 10 Years On*. Geneva, Switzerland: World Health Organization.

[30] Jadotte E, 2012*. Brain Drain, Brain Circulation and Diaspora Networks in Haiti. United Nations Conference on Trade and Development, The Least Developed Countries Report 2012* Available at: http://unctad.org/en/PublicationsLibrary/ldcr2012-bp1.pdf.

[31] Kristof M Panarelli L, 2010. Haiti: A Republic of NGO’s? U.S. Institute of Peace.

[32] World Health Organization, 2014. *World Health Statistics*. Geneva, Switzerland: WHO.

[33] Sands P Mundaca-Shah C Dzau VJ, 2016 The neglected dimension of global security: a framework for countering infectious-disease crises. N Eng J Med 374: 1281–1287. 10.1056/NEJMsr1600236 26761419

[33] Kligerman M Walmer D Merrell SB, 2015 The socioeconomic impact of international aid: a qualitative study of healthcare recovery in post-earthquake Haiti and implications for future disaster relief. Glob Pub Health 12: 531–544. 10.1080/17441692.2015.1094111 26565063

[35] Engebretsen E Heggen K Das S Farmer P Otterson P, 2016 Paradoxes of sustainability with consequences for health. Lancet 4: e225–e226. 27013304 10.1016/S2214-109X(16)00038-3

